# Whole-exome sequencing in a subject with fluctuating neuropsychiatric symptoms, immunoglobulin G1 deficiency, and subsequent development of Crohn’s disease: a case report

**DOI:** 10.1186/s13256-022-03404-9

**Published:** 2022-05-11

**Authors:** Harumi Jyonouchi, Lee Geng

**Affiliations:** 1 Department of Pediatrics, Saint Peter’s University Hospital (SPUH), New Brunswick, USA; 2 Department of Pediatrics, Rutgers University-Robert Wood Johnson Medical School, SPUH, 254 Easton Ave., New Brunswick, NJ 08901 USA

**Keywords:** Whole-exome sequencing, Crohn’s disease, *NLRP12*, *IRF2BP2*, Interleukin-1β, Tumor necrosis factor-α, Pediatric acute-onset neuropsychiatric syndrome

## Abstract

**Background:**

Mutations or polymorphisms of genes that are associated with inflammasome functions are known to predispose individuals to Crohn’s disease and likely affect clinical presentations and responses to therapeutic agents in patients with Crohn’s disease. The presence of additional gene mutations/polymorphisms that can modify immune responses may further affect clinical features, making diagnosis and management of Crohn’s disease even more challenging. Whole-exome sequencing is expected to be instrumental in understanding atypical presentations of Crohn’s disease and the selection of therapeutic measures, especially when multiple gene mutations/polymorphisms affect patients with Crohn’s disease.

**Case summary:**

We report the case of a non-Hispanic Caucasian female patient with Crohn’s disease who was initially diagnosed with pediatric acute-onset neuropsychiatric syndrome with fluctuating anxiety symptoms at 9 years of age. This patient was initially managed with pulse oral corticosteroid treatment and then intravenous immunoglobulin due to her immunoglobulin G1 deficiency. At 15 years of age, she was diagnosed with Crohn’s disease, following onset of acute abdomen. Treatment with oral corticosteroid and then tumor necrosis factor-α blockers (adalimumab and infliximab) led to remission of Crohn’s disease. However, she continued to suffer from chronic abdominal pain, persistent headache, general fatigue, and joint ache involving multiple joints. Extensive gastrointestinal workup was unrevealing, but whole-exome sequencing identified two autosomal dominant gene variants: *NLRP12* (loss of function) and *IRF2BP2* (gain of function). Based on whole-exome sequencing findings, infliximab was discontinued and anakinra, an interleukin-1β blocker, was started, rendering marked improvement of her clinical symptoms. However, Crohn’s disease lesions recurred following *Yersinia* enterocolitis. The patient was successfully treated with a blocker of interleukin-12p40 (ustekinumab), and anakinra was discontinued following remission of her Crohn’s disease lesions.

**Conclusion:**

Loss-of-function mutation of *NRLRP12* gene augments production of interleukin-1β and tumor necrosis factor-α, while gain-of-function mutation of *IRF2BP2* impairs cytokine production and B cell differentiation. We propose that the presence of these two autosomal dominant variants caused an atypical clinical presentation of Crohn’s disease.

## Introduction

Inflammasomes are cytosolic protein complexes involved in innate immune responses, sensing nonspecific signals through pattern recognition receptors that belong to a nucleotide-binding oligomerization domain-like receptor (NLR) family [1]. Upon these signals, inflammasomes convert pro-interleukin (IL)-1β and IL-8 into active forms and release them. Most NLR family members are associated with inflammasomes that promote inflammatory responses, while NLR protein 12 (NLRP12) exerts suppressive actions [1]. Autosomal dominant LOF mutations of *NLRP12* cause familial autoinflammatory syndrome [2]. NLRP12 also plays a role in intestinal homeostasis, in part promoting bacterial tolerance in the gut [3]. In rodent models, NLRP12 deficiency leads to colitis caused by an increase in production of TNF-α and IL-6 [3]. LOF *NLRP12* mutations were reported to cause autoinflammatory syndrome [2]. In patients with inflammatory bowel disease (IBDs), polymorphism of *NLRP12* is associated with changes in TNF-α production by peripheral blood mononuclear cells (PBMCs) [4]. Therefore, individuals with LOF *NLRP12* variants are likely prone to IBD and may present with symptoms observed in patients with autoinflammatory syndromes.

The availability of WES began to disclose that the presence of additional mutations can alter the clinical presentation of *NLRP12* variants. Tal *et al.* reported a case of *NLRP12* mutation with a variant of toll-like receptor 3 (TLR3) which presented as severe esophagitis of human simplex virus (HSV) and CD without periodic fever [5]. A child with variants of *POLR3A* and *NLRP12* reportedly presented with recurrent acute disseminated encephalomyelitis/optic neuritis along with familial cold autoinflammatory syndrome [6].

The presented case was initially diagnosed with PANS, with favorable responses to pulse OCS and then IVIg. However, the patient subsequently developed CD and revealed atypical responses to TNF-α blockers. WES identified AD variants of *NLRP12* and *IRF2BP2*. A GOF mutation of *IRF2BP2* is reported in familial common variable immunodeficiency (CVID), resulting in impairment of B cell differentiation, and production of T cell/monocyte cytokines. In the presented case, the presence of these two variants appears to have resulted in a puzzling array of clinical features.

## Case presentation

The presented case is a non-Hispanic Caucasian female who was diagnosed with PANS at age 9 years, following acute onset of severe anxiety that manifested as separation anxiety and school phobia. Tics, and other physical symptoms typically seen in PANS patients, were not present as per her parents. The patient was initially treated with ibuprofen, and then OCS. The patient responded favorably to pulse OCS therapy initially. Secondary to persistently low immunoglobulin 1 (IgG1), the patient was referred to the Pediatric Allergy/Immunology Clinic at age 10 years. Her condition was stable for the next 2 years with prophylaxis antibiosis (azithromycin) only. At age 12 years, following a viral syndrome, a major exacerbation of anxiety occurred and a high dose IVIg (1 gram/kg/dose x2) caused a severe adverse reaction (nausea, vomiting, and severe headache). Supplemental IVIg (0.8 g/kg/dose every 3 weeks) to prevent recurrent respiratory infection was well tolerated, with resolution of her anxiety symptoms. However, at age 15 years, sudden onset of acute abdomen resulted in a visit to the emergency room. Abdominal pain progressively worsened over the next several months. She was eventually diagnosed with CD based on biopsy findings: CD lesions in the terminal ileum. Her CD was treated with oral budesonide (Entocort EC) for 1 month without improvement. Thus, adalimumab 7.5 mg subcutaneous (SQ) injection every 2 weeks was started, and then switched to 7. 5 mg SQ every week, resulting in improvement of her gastrointestinal (GI) symptoms with resolution of CD lesions 8 months after weekly adalimumab 7.5 mg SQ. However, she suffered from severe joint symptoms; she was found to have positive antihistone antibody and thought to have had drug-induced lupus. Therefore, adalimumab was switched to infliximab IV (300 mg/dose) every 8 weeks and then eventually ever 4 weeks. Switching to infliximab IV infusion led to onset of severe fatigue/headache and adverse reactions to IVIg (joint ache, headache, skin rash, and malaise). Part of her pain symptoms were attributed to subcutaneous nerve entrapment at that time. Prior to the start of TNF-α blockers, she tolerated IVIg without any adverse reactions. During the next 12 months, she continued to have severe fatigue, headache, joint ache, and recurrence of abdominal pain without symptomatic relief by changing Ig infusion from IV to SQ routes. Various regimens of pre- and post-medications for IVIg provided no symptomatic relief. The results of extensive GI workup exploring the organic causes affecting intestine, pancreas, and liver were unrevealing. Because of her puzzling clinical features, we turned to genetic analysis. WES revealed AD variants of *NLRP12* [C.1054>T, minor allele frequency (MAF) 0.04%] and *IRF2BP2* (c.1180A>C, MAF 0.006%), and heterozygous, autosomal recessive, pathological variant of *ATM* (c.7089+2T>G). GOF mutations of *IRF2BP2* were reported to cause hypogammaglobulinemia, suppressing production of cytokines and B cell differentiation [7]. NLRP12 is reported to have a role in gut immune homeostasis, and the mutation found in this case is reported in two patients with cryopyrin-associated periodic fever syndrome (CAPS) [8]. These variants are inherited from her father. Interestingly, the paternal grandmother, who was diagnosed with rheumatoid arthritis, did not respond well to TNF-α inhibitors either, as per her parents. Prior to this genetic workup, the presented case was diagnosed with low Von Willebrand status based on DDVAP challenge in 2017; she has had symptoms of easy bruising and hypermenorrhea.

Given the similarity of her neuropsychiatric and joint symptoms to autoinflammatory syndrome and her lack of responses to TNF-α inhibitors, infliximab was discontinued. Then, 10 weeks after, anakinra, an IL-1β blocker, was started (100 mg/day). Supplemental Ig was also discontinued secondary to her severe adverse reactions. She revealed marked improvement of her clinical symptoms (headache, fatigue, joint ache, sleep disturbance, and symptoms resembling postural orthostatic tachycardia syndrome (POTS)) over 2–3 months after starting anakinra SQ injection. However, her POTS symptoms were not completely resolved. Five months after starting anakinra, she had recurrence of mild diarrhea that resolved with a 10-day course of metronidazole, although microbial workup was negative. Then, 12 months after discontinuation of infliximab, the GI symptoms due to *Yersinia* enterocolitis occurred, which led to the recurrence of CD lesions. Thus, 5 months after onset of *Yersinia* enterocolitis, treatment with ustekinumab (45 mg SQ injection x2, 4 weeks apart, then every 12 weeks) was initiated, and remission of CD was achieved in 6 months. Anakinra was discontinued after remission was achieved.

## Laboratory findings

Two AD variants of *NLRP12* and *IRF2BP2* are expected to alter cytokine production by monocyte–macrophage lineage cells [9–11]. Therefore, cytokine production by purified peripheral blood monocytes (PBMo) with methodologies reported elsewhere [12] was determined at two time points: prior to starting anakinra, which was 2 months after discontinuation of infliximab, and then 5 months after starting anakinra.

**Fig. 1:**
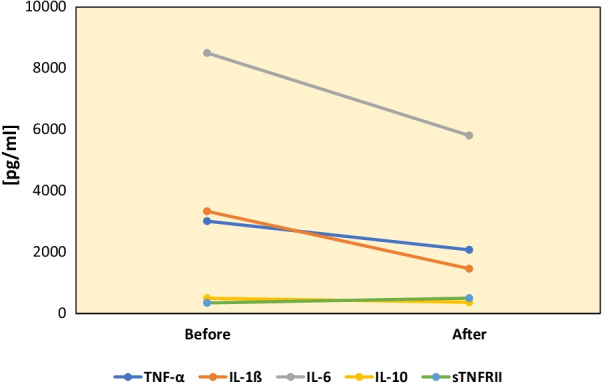
Changes of cytokine levels produced before and 5 months after starting anakinra 100 mg SQ injection daily

The results of these assays revealed a decrease in the production of IL-1β, IL-6, and TNF-α in response to candida heat extract as a source of β-glucan, which activates the inflammasome pathway [12]. There was no increase in spontaneous production of cytokines at two time points. Production of counterregulatory cytokines (IL-10, sTNFRII, or TGF-β) in response to β-glucan did not differ after starting anakinra (Fig. 1). Such changes were not observed in PBMo cytokine production in response to TLR agonists (LPS, zymosan, and CL097). Production of IL-1β and TNF-α was evaluated after initiation of usterkinumab treatment, following the initial submission of this manuscript. They remained within normal limits.

## Discussion

Progress in genetic analysis has made it possible to evaluate the effects of variants of genotypes on both the clinical manifestation and therapeutic responses of autoimmune diseases. Risk variants of genes have been implicated in the onset and progression of IBD, in close association to interactions with gut microbiota [13]. The presented case illustrates complex gene–gene interactions in association with IBD and the utility of WES for assessing atypical clinical manifestation of IBD.

NLRP12 has emerged as a crucial regulator of innate immunity, and the presence of NLRP12 variants is expected to increase the risk of GI inflammation by affecting gut immune homeostasis. In rodent models, NLRP12 is reported to promote bacterial tolerance, augmenting the diversity of microbe and gut immune homeostasis [3]. This is partly due to proteasomal degradation of NOD2 by NLRP12 [10]. NLRP12 is also known to exert a broad range of regulatory actions on major activation signaling pathways (NF-kB, NFAT, MAPK, and mTOR pathways) [14]. As a result, the presence of NLRP12 variants is expected to result in increased production of inflammatory cytokines, which include IL-1β, IL-6, and TNF-α. In IBD patients, increase in serum levels of TNF-α is associated with the *NLRP12* variant (rs34436714) [4]. In two CAPS patients, with the same NLRP12 variant that was found in the presented case, increase in spontaneous production of TNF-α and IL-1β has been reported [9]. Therefore, presence of the *NLRP12* variant in the presented case is expected to be a predisposing factor for developing IBD.

The increased production of inflammatory cytokines, discussed above, is also expected to affect the central nervous system. It is well known that patients with dysregulated cytokine production of IL-1β as seen in autoinflammatory syndromes are known to exhibit neuropsychiatric symptoms [15]. Increase in inflammatory cytokines (IL-1β, IL-6, and TNF-α) has also be reported in common neuropsychiatric conditions including depression and schizophrenia [16]. It is of note that the presented case was initially diagnosed with PANS due to predominant anxiety symptoms, although she did not reveal other symptoms typically seen in PANS (tics, changes in fine motor functions, etc.). Increase in these inflammatory cytokines and the resultant symptoms can be controlled with OCS. In fact, in the presented case, initially, she responded well to pulse OCS for controlling anxiety.

WES analysis revealed that she had an AD GOF variant of *IRF2BP2*. IRFBP2 exerts regulatory functions on multiple lineage cells, by serving as a transcriptional co-repressor [17–19]. These actions of IRF2BP2 on monocyte–macrophage lineage cells suppress the differentiation of inflammatory type 1 (M1) macrophages, attenuating inflammation [11, 20]. Repressive actions of IRF2BP2 have been shown to attenuate atherosclerosis and CN inflammation, partly suppressing production of inflammatory cytokines [11, 20]. However, excessive suppression through IRF2BP2 can be harmful. It was shown that an AD GOF mutation of *IRF2BP2* was implicated in a familial form of common variable immunodeficiency (CVID) [7]. In the reported CVID cases with the *IRF2BP2* mutation, excessive IRF2BP2 actions impaired terminal differentiation of B lineage cells [7].

The presented case paternally inherited AD variants of both *NLRP12* and *IRF2BP2*. A LOF AD variant of *NLRP12* is expected to augment production of inflammatory cytokines and disrupt gut immune homeostasis, while the AD GOF variant of *IRF2BP2* suppresses activation signals of multiple lineage cells. We hypothesized that the presence of the AD *NLRP12* variant predisposed her to CD and neuropsychiatric symptoms following immune insults. Development of hypogammaglobulinemia may have been associated with the AD GOF *IRF2BP2* variant. Antagonistic actions of these two variants (*IRF2BP2* and *NLRP12*) may have provided the relatively stable condition seen during treatment with monthly IVIg, which likely attenuated immune insults from microbial infection. However, the development of CD and subsequent treatment with TNF-α inhibitors may have augmented the effects of the *NLRP12* variant on inflammasomes, leading to clinical features resembling autoinflammatory syndromes. Lack of recurrent fever and skin symptoms may have been associated with the GOF *IRF2BP2* variant. Once CD reached remission, we hypothesized that a low dose of anakinra (100 mg/day) may have helped restore balance to her inflammatory responses. Indeed, she revealed marked improvement of her clinical symptoms with anakinra. In two patients with the same *NLRP12* variant as found in the presented case, anakinra treatment caused increase in TNF-α production within 3–4 months [9]. However, in the presented case, anakinra treatment did not cause an increase in TNF-α production (Fig 1). In fact, this patient did not reveal an increase in spontaneous production of TNF-α or IL-1β. This may be due to the presence of the GOF *IRF2BP2* variant. However, following immune insults, she may require additional anti-inflammatory medications to control TNF-α-mediated inflammation. This became apparent when she experienced recurrence of CD lesions following *Yersinia* enterocolitis. Treatment with ustekinumab was successful for controlling her recurrent CD lesions and led to the subsequent discontinuation of anakinra. This may be due to the fact that ustekinumab, a monocloncal antibody against IL-12p40, which blocks signaling pathways of IL-12 and IL-23, can successfully block IL-1β-mediated Th17 cell activation as well as TNF-α-induced Th1 activation [21, 22].

However, her fluctuating neuropsychiatric symptoms may also be affected by other factors. For example, the presence of maternal diabetes and use of steroids can worsen the manifestation of neuropsychiatric symptoms in Crohn’s disease [23, 24], although her mother denied history of gestational diabetes during her pregnancy.

## Conclusions

The revelation of two AD GOF gene variants by WES provided a better understanding of the atypical clinical features and failed responses to TNF-α inhibitors in the presented case. In IBD cases with puzzling clinical features and suboptimal responses to first.line therapeutic measures, genetic workup using WES may be helpful. In addition, her atypical course of CD emphasizes the importance of endoscopic biopsies for diagnosis and follow-up.

## Data Availability

The datasets used and/or analyzed during the current study are available from the corresponding author on reasonable request.

## Abbreviations

AD: Autosomal dominant
CAPS: Cryopyrin-associated periodic fever syndrome
CD: Crohn’s disease
CVID: Common variable immunodeficiency
GOF: Gain of function
IBD: Inflammatory bowel disease
Ig: Immunoglobulin
IL: Interleukin
IVIg: Intravenous immunoglobulin
LOF: Loss of function
LPS: Lipopolysaccharide
MAF: Minor allele frequency
NLR: NOD-like receptor
OCS: Oral corticosteroids
PANS: Pediatric acute-onset neuropsychiatric syndrome
PBMo: Peripheral blood monocytes
SQ: Subcutaneous
TLR: Toll-like receptor
TNF: Tumor necrosis factor
WES: Whole-exome sequencing
